# Supplementary material for: Identification of photoperiod-induced specific miRNAs in the adrenal glands of Sunite sheep (Ovis aries)
Source: Front Vet Sci. 2022 Jul 22;9:888207. doi: 10.3389/fvets.2022.888207 (PMC9354845; doi:10.3389/fvets.2022.888207)
Supplement: Supplementary file 1 [file Data_Sheet_1.ZIP › Supplementary file/miRDeep2 file.zip!/miRDeep2 file/Novel_Structure/SP42A3/Novel_249.pdf]

Provisional ID	:	NC_019463.2_11853
Score total	:	2.3
Score for star read(s)	:	-1.3
Score for read counts	:	0
Score for mfe	:	2
Score for randfold	:	1.6
Score for cons. seed	:	
Total read count	:	356
Mature read count	:	356
Loop read count	:	0
Star read count	:	0

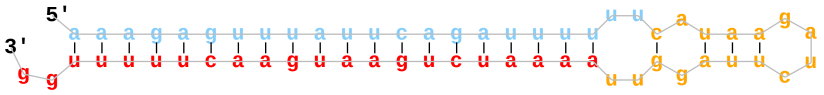

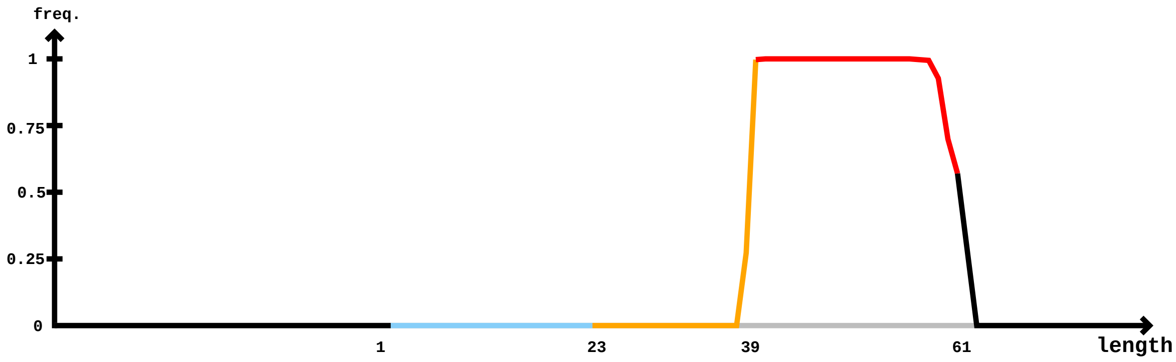

Star **Mature**

```
5'- uacuauuaacucuaaauaguuaauagguuggccaaagaguuuauucagauuuuuucauaagaucuuagguuaaaaucugaaugaacuuuuuggccaaguuacuaauuuaauu   -3'    exp
    ..(((((((((......))))))))).((((((((((((((((((((((((((..(.(((....))).)..))))))))))))))))))))))))))...............    reads       mm         sample
    ......................................................................Aaaaaucugaaugaacuu........................    1           1          seq
    ......................................................................Gaaaaucugaaugaacuuu.......................    1           1          seq
    ......................................................................Aaaaaucugaaugaacuuuu......................    24          1          seq
    ......................................................................Aaaaaucugaaugaacuuuuu.....................    69          1          seq
    ......................................................................Gaaaaucugaaugaacuuuuu.....................    1           1          seq
    ......................................................................uaaaaucugaaugaacuuuuug....................    1           0          seq
    .......................................................................aaaaucugaaugaaUuuuuu.....................    1           1          seq
    .......................................................................aaaCucugaaugaacuuuuu.....................    7           1          seq
    .......................................................................aaaaucugaaugaacuuuCu.....................    1           1          seq
    .......................................................................aaaaucugaauAaacuuuuu.....................    1           1          seq
    .......................................................................aaaaucugaaugGacuuuuu.....................    1           1          seq
    .......................................................................aaaaucugaaugaacuuuuuC....................    1           1          seq
    .......................................................................aaaaucugaaugaacuuuuug....................    44          0          seq
    .......................................................................aaaaucuAaaugaacuuuuugg...................    1           1          seq
    .......................................................................aaaaucugaaugaacuuuuugg...................    191         0          seq
    .......................................................................aaaaucugaaugaacuuuuuggU..................    8           1          seq
    .......................................................................aaaaucugaaugaacuuuuuggc..................    2           0          seq
    ........................................................................aaaucugaaugaacuuuuugg...................    1           0          seq
```
